# Upregulation of SOX9 in osteosarcoma and its association with tumor progression and patients’ prognosis

**DOI:** 10.1186/1746-1596-8-183

**Published:** 2013-11-04

**Authors:** Haibo Zhu, Jie Tang, Mingjie Tang, Haikang Cai

**Affiliations:** 1 Orthopaedics Department, Xuhui central hospital, No. 966, Middle Huaihai Road, Shanghai 200031, China; 2 Orthopaedics Department, Shanghai Sixth people's hospital, Shanghai Jiaotong University, Shanghai 200233, China

**Keywords:** Osteosarcoma, SOX9, Prognosis, Overall survival, Disease-free survival

## Abstract

**Objective:**

SOX9 plays an important role in bone formation and tumorigenesis. However, its involvement in osteosarcoma is still unclear. The aim of this study was to investigate the expression pattern and the clinical significance of SOX9 in human osteosarcoma.

**Methods:**

SOX9 mRNA and protein expression levels were detected by RT-PCR and Western blot assays, respectively, using 30 pairs of osteosarcoma and noncancerous bone tissues. Then, immunohistochemistry was performed to analyze the association of SOX9 expression in 166 osteosarcoma tissues with clinicopathological factors or survival of patients.

**Results:**

SOX9 expression at mRNA and protein levels were both significantly higher in osteosarcoma tissues than those in corresponding noncancerous bone tissues (both P < 0.001). Immunohistochemical staining indicated that SOX9 localized to the nucleus and high SOX9 expression was observed in 120 of 166 (72.3%) osteosarcoma specimens. In addition, high SOX9 expression was more frequently occurred in osteosarcoma tissues with advanced clinical stage (P = 0.02), positive distant metastasis (P = 0.008) and poor response to chemotherapy (P = 0.02). Osteosarcoma patients with high SOX9 expression had shorter overall survival and disease-free survival (both P < 0.001). Furthermore, the multivariate analysis confirmed that upregulation of SOX9 was an independent and significant prognostic factor to predict poor overall survival and disease-free survival (both P = 0.006).

**Conclusions:**

Our data show for the first time that SOX9 is upregulated in aggressive osteosarcoma tissues indicating that SOX9 may participate in the osteosarcoma progression. More importantly, SOX9 status is a useful prognostic factor for predicting the prognosis of osteosarcoma, suggesting that SOX9 may contribute to the optimization of clinical treatments for osteosarcoma patients.

**Virtual slides:**

The virtual slides for this article can be found here: http://www.diagnosticpathology.diagnomx.eu/vs/1318085636110837.

## Introduction

Osteosarcoma, a kind of highly aggressive neoplasm arising from long bones, is the most common primary malignancy in children and adolescents [1]. Although it is a relative uncommon cancer with an estimated worldwide incidence of 2 ~ 3 cases per million persons per year, osteosarcoma predominately arises in bone during periods of rapid growth and has a predilection to affect adolescents and young adults, and if untreated it is fatal [2]. Characteristically, osteosarcoma often occurs from low-grade lesions with low malignant potential, through to high-grade lesions with the potential for distant metastasis, and it metastasizes preferentially to the lung [3]. Despite recent advances in modern treatment protocols combining chemotherapy, surgery, and sometimes radiotherapy, the 5-year overall survival and disease-free survival rates for patients with osteosarcoma are around 50-60% [4]. In order to give effective treatment to each patient, accumulating studies have tried to predict the clinical course of osteosarcoma patients by assessing the response to chemotherapy histologically or by determining clinical stage. However, these features have limited utility in terms of predicting the clinical course of osteosarcoma patients, which suggests that different genetic mechanisms may be operating and altering the response to chemotherapy and metastatic capability of osteosarcomas with the same clinicopathological features [5]. Therefore, molecular pathogenesis of osteosarcoma, which can identify tumor pathways and specific mediators, should be clarified in order to provide insight into tumorigenesis and tumor progression, and subsequently develop novel approaches for treating osteosarcoma.

As a developmental transcription factor, Sex determining region Y (SRY)-related high mobility group (HMG)-box 9 (SOX9) plays a vital role in the regulation of sex determination, cartilage development, intestinal differentiation and adult progenitor cell pool maintenance [6]. SOX9 shares 70% amino acid homology to SRY through its HMG box, the domains of which are involved in the regulation of DNA-dependent processes, such as transcription and replication [7]. In humans, SOX9 heterozygous mutations result in campomelic dysplasia, a syndrome characterized by severe skeletal malformations, defects in the central nervous system and several other organs, frequent XY female sex reversal, and perinatal lethality [8]. Duplications of the *SOX9* gene or its deliberate mis-expression have been linked with XX male sex reversal and fibrosis-related disorders and suggest that dysregulation of the gene can cause disease. In particular, recent studies have indicated the emerging role of SOX9 in various human cancers. SOX9 has been found to be overexpressed in gastric carcinoma, non-small cell lung cancer, prostate cancer, breast cancer, pancreatic ductal adenocarcinoma, glioma, colorectal cancer and ovarian cancer [9-16]. In contrast, SOX9 may function as a tumor suppressor, at least in some melanomas [17]. These findings suggest that SOX9 may play different roles in various types of malignancies. However, very little is known about SOX9 in human osteosarcoma. Since it has been demonstrated to be an important regulator of the bone development and chondrocyte phenotype, we hypothesized that SOX9 may be a candidate marker for osteosarcoma progression. In the current study, we conducted RT-PCR, Western blot and immunohistochemistry assays to determine the expression patterns of SOX9 at both mRNA and protein levels in osteosarcoma tissues. We further revealed the clinical significance of the aberrant expression of SOX9 in this disease.

## Materials and methods

### Patients and tissue samples

This study was approved by the Research Ethics Committee of Shanghai Sixth people's hospital, Shanghai Changhai hospital, Shanghai East hospital, Zhujiang Hospital, and Xuhui central hospital, China. Written informed consent was obtained from all of the patients. All specimens were handled and made anonymous according to the ethical and legal standards.

For RT-PCR and Western blot assays, 30 primary osteosarcoma and corresponding noncancerous bone tissue samples from the same specimens were immediately frozen in liquid nitrogen and stored at -80°C until use. For immunohistochemistry analysis, 166 primary osteosarcoma and corresponding noncancerous bone tissue samples from the same specimens were collected from the Department of Pathology, Shanghai Sixth people's hospital, Shanghai Changhai hospital, Shanghai East hospital, Zhujiang Hospital, and Xuhui central hospital, China, from January 1998 to March 2008. No patients had received blood transfusion, radiotherapy, or chemotherapy before surgery. Clinical stage of these osteosarcoma patients were classified according to the sixth edition of the tumor-node-metastases (TNM) classification of the International Union against Cancer (UICC). The clinicopathological information of the patients is shown in Table 1. Immunostainings were performed on archived paraffin wax embedded biopsy specimens.

**Table 1:** Correlation of SOX9 expression with clinicopathological features of osteosarcoma

**Clinicopathological features**	**No.of cases**	**SOX9 expression**		**P**
		**High(n, %)**	**Low(n, %)**	
**Age**				
<55	72	50 (69.4)	22 (30.6)	NS
≥55	94	70 (74.5)	24 (25.5)	
**Gender**				
Male	96	70 (72.9)	26 (27.1)	NS
Female	70	50 (71.4)	20 (28.6)	
**Tumor size(cm)**				
>8	88	62 (70.5)	26 (29.5)	NS
≤8	78	58 (74.4)	20 (25.6)	
**Anatomic location**				
Tibia/femur	103	76 (73.8)	27 (26.2)	NS
Elsewhere	63	44 (69.8)	19 (30.2)	
**Serum level of lactate dehydrogenase**				
Elevated	90	70 (77.8)	20 (22.2)	NS
Normal	76	50 (65.8)	26 (34.2)	
**Serum level of alkaline phosphatase**				
Elevated	108	80 (74.1)	28 (25.9)	NS
Normal	58	40 (69.1)	18 (30.9)	
**Clinical stage**				
IIA	68	32 (47.1)	36 (52.9)	0.02
IIB/III	98	88 (89.8)	10 (10.2)	
**Distant metastasis**				
Absent	124	78 (62.9)	46 (37.1)	0.008
Present	42	42 (100.0)	0 (0)	
**Response to chemotherapy**				
Good	68	32 (47.1)	36 (52.9)	0.02
Poor	98	88 (89.8)	10 (10.2)	

All 166 osteosarcoma patients received follow-up. The median follow-up was 87 months (range: 10–152 months). During the follow-up period, 66 patients (66/166, 39.8%) died of disease. Distant metastases developed in 42 patients at a mean of 13.8 months (range 3–46 months) after the original diagnosis. Of these patients, 9 had bone metastases and 36 had lung metastases (3 patients had both bone and lung metastases). The median overall and disease-free survival of patients was 31 months (95% confidence interval [CI], 30.1-42.9 months) and 25 months (95% CI, 23.7-35.2 months), respectively.

### Real-time quantitative RT-PCR Assay

The expression levels of SOX9 mRNA in osteosarcoma and corresponding noncancerous tissues were detected by real-time quantitative RT-PCR assay according to our previous study [18]. Briefly, total RNA was extracted from tissues using TRIzol (Invitrogen, Carlsbad, CA) according to the manufacturer’s protocol. The reverse transcription reaction was performed using high-capacity cDNA synthesis kit (Applied Biosystems, Foster City, CA). Equal cDNA amounts from each sample were amplified using the following primers: for human SOX9, forward primer, 5'- CGA AAT CAA CGA GAA ACT GGA C -3', and reverse primer, 5'- ATT TAG CAC ACT GAT CAC ACG -3'; for human glyceraldehyde 3-phosphate dehydrogenase (GAPDH), forward primer, 5'- CCC ACT CCT CCA CCT TTG AC-3', and reverse primer, 5'-ATG AGG TCC ACC ACC CTG TT-3'. Each sample was examined in triplicate and the amounts of the PCR products produced were non-neoplasticized to GAPDH which served as internal control.

### Western blot analysis

The expression levels of SOX9 protein in osteosarcoma and corresponding noncancerous bone tissues were detected by Western blot analysis according to our previous study [18]. Briefly, the tissues were harvested by scraping and lysed using modified radio immunoprecipitation assay buffer (50 mM Tris–HCl, pH 7.4), 1% NP-40, 0.25% sodium deoxycholate, 150 mM NaCl, 1 mM ethylenediaminetetraacetic acid (EDTA), protease inhibitor cocktail complete. The protein content was determined according to Bradford’s method. Equal protein amounts from each sample were separated electrophoretically on 7.5% SDS-polyacrylamide gels and transferred onto polyvinylidene difluoride membranes (Roche; Basel, Switzerland). The membranes were probed with monoclonal mouse antihuman SOX9 (sc-166505, 1:200, Santa Cruz Biotechnology, Santa Cruz, CA, USA). The expression level of SOX9 was determined by incubating the membranes with horseradish peroxidase-conjugated anti-mouse immunoglobulin G (1:3000 dilution) and enhanced chemiluminescence reagent (Pierce; Minneapolis, MN, USA) according to the manufacturer’s protocol. Anti-β-actin mouse monoclonal antibody (1:1000 dilution: Sigma; St Louis, MO, USA) was used as a loading control. Each sample was examined in triplicate and the expression level of SOX9 protein was evaluated as an optical densitometry (OD) ratio that was scored as the densitometry of SOX9 relative to the densitometry of β-actin.

### Immunohistochemistry analysis

The sub-cellular localization and expression levels of SOX9 protein in osteosarcoma and corresponding noncancerous bone tissues were detected by immunohistochemistry analysis using paraffin-embedded specimens from 166 patients with osteosarcoma according to our previous study [18]. Briefly, the paraffin-embedded tissues were cut at 3μm and stained following being dried on ProbeOn Plus (Fisher Scientific International, Hampton, NH, USA). The slide to which a paraffin section was attached went through deparaffinization and hydration, and was then treated with a solution of Peroxidase-blocking reagent (DAKO, Hamburg, Germany) to exhaust endogenous peroxidase activity. To inhibit non-specific antigen-antibody reactions possible in immunohistochemical staining, reaction was done using a protein blocker (Research Genetics, Huntsville, AL, USA) for 5 min and the slide was washed thoroughly with water. The slides were incubated overnight with the primary SOX9 antibody (mouse monoclonal antibody, sc-166505, 1:200, Santa Cruz Biotechnology, Santa Cruz, CA, USA) at 4°C. Secondary antibody for the detection of primary antibody was reacted for 10 min using anti-mouse IgG (Sigma, St. Louis, MO, USA) to which biotin was attached, and then washed with buffer solution and reacted with horseradish peroxidase for 10 min. After hematoxylin contrast staining, the slide was enclosed with Universal Mount (Research Genetics) and examined. In each immunohistochemistry run, noncancerous bone tissues were used as control tissues and non-immune IgG was also used as negative control antibody for immunohistochemical staining.

Assessment of immunohistochemical staining was evaluated by two independent pathologists who were blinded to the clinicopathological parameters and clinical outcomes of the patients. The SOX9-positive cells showed immunoreactivity in the nucleus of tumor cells. The number of SOX9 positive-staining cells showing immunoreactivity in ten representative microscopic fields was counted and the percentage of positive cells was calculated. The percentage scoring of immunoreactive tumor cells was as follows: 0 (0%), 1 (1-10%), 2 (11-50%) and 3 (>50%). The staining intensity was visually scored and stratified as follows: 0 (negative), 1 (weak), 2 (moderate) and 3 (strong). A final immunoreactivity scores (IRS) was obtained for each case by multiplying the percentage and the intensity score. The median of IRS for all examined samples was 5.0. Thus, the SOX9 protein expression levels were further analyzed by classifying IRS values as low (based on a IRS value less than 5.0) and as high (based on a IRS value greater than 5.0).

### Statistical analysis

The software of SPSS version13.0 for Windows (SPSS Inc, IL, USA) and SAS 9.1 (SAS Institute, Cary, NC) was used for statistical analysis. Continuous variables were expressed as $\bar{X}\pm s$. Spearman rank correlation is used to analyze the correlation between SOX9 mRNA expression and SOX9 protein expression. The Chi-square test was used to show differences of categorical variables. Patient survival and their differences were determined by Kaplan–Meier method and log-rank test. Cox regression (Proportional hazard model) was adopted for multivariate analysis of prognostic factors. Differences were considered statistically significant when *P* was less than 0.05.

## Results

### Upregulation of SOX9 at mRNA and protein levels in human osteosarcoma tissues

**Figure 1:**
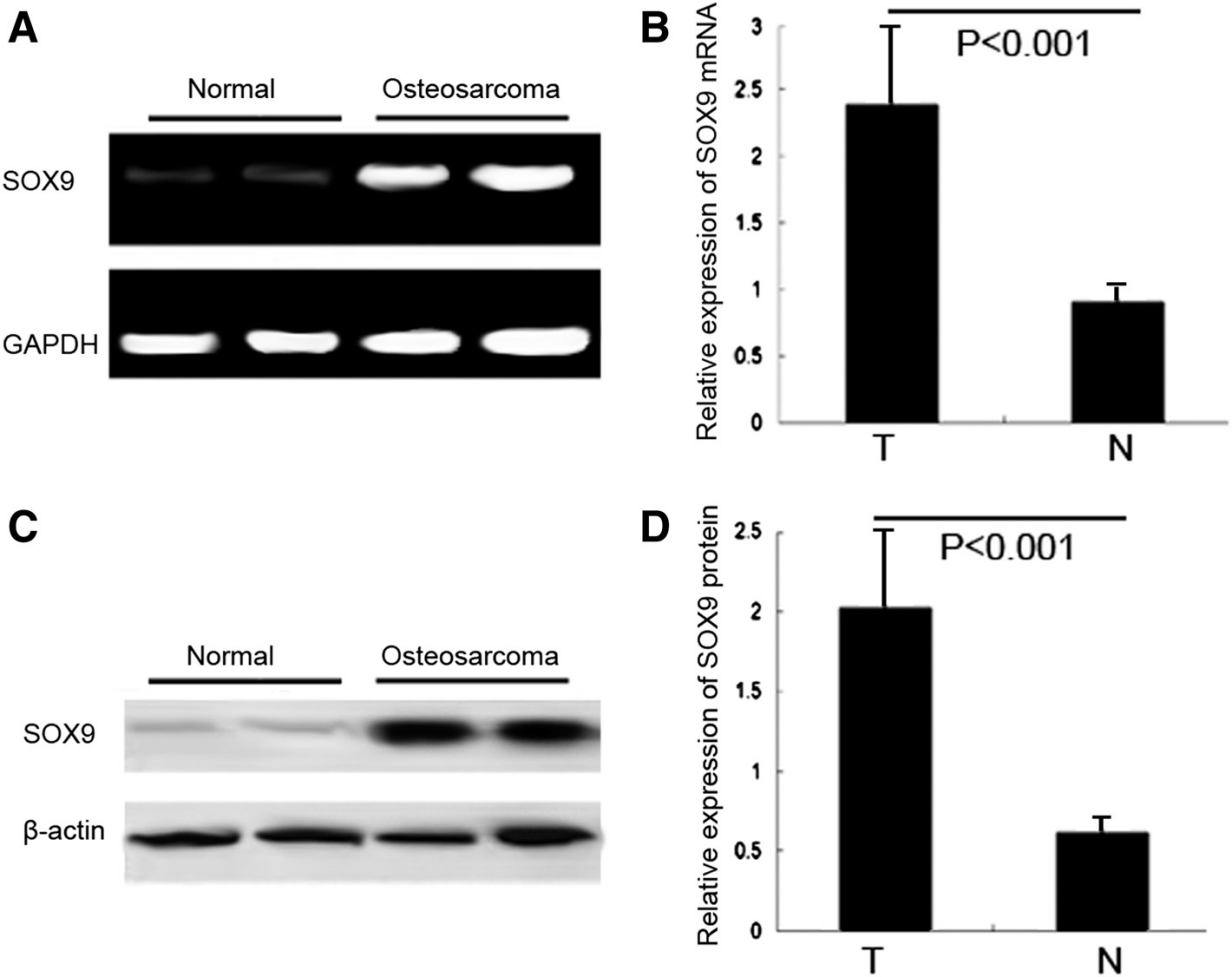
**SOX9 mRNA and protein expression in 30 osteosarcoma and corresponding noncancerous bone tissues were respectively detected by real**-**time quantitative RT**-**PCR assay and Western blot analysis. (A)** Expression levels of SOX9 mRNA in osteosarcoma tissues and noncancerous bone tissues. **((B)** A graphical representation of the SOX9 mRNA level expression profiles in **A)**. **(C)** Expression levels of SOX9 protein in osteosarcoma tissues and noncancerous bone tissues. **(D)** A graphical representation of the SOX9 protein level expression profiles in **(C)**. 'N' refers to noncancerous bone tissues; 'T' refers to osteosarcoma tissues.

As shown in Figure 1A, the expression levels of SOX9 mRNA were found to be distinctly increased in osteosarcoma tissues compared to noncancerous bone tissues. The statistic analysis showed that the relative level of SOX9 mRNA expression in osteosarcoma tissues (mean ± SD: 2.4 ± 0.6) was significantly higher than that in corresponding noncancerous bone tissues (mean ± SD: 0.9 ± 0.1; P < 0.001, Figure 1B). Similarly, the relative expression levels of SOX9 protein in osteosarcoma tissues (mean ± SD: 2.1 ± 0.4) tended to be significantly higher than that in corresponding noncancerous bone tissues (mean ± SD: 0.6 ± 0.08; P < 0.001; Figure 1C and D). More importantly, there was a significant correlation between SOX9 mRNA expression and SOX9 protein expression (r = 0.8; P < 0.001; Spearman rank correlation analysis).

### Upregulation of SOX9 protein associates with advanced clinicopathological features of osteosarcoma

SOX9 positive staining was localized in the cell nucleus in primary osteosarcoma and corresponding noncancerous bone tissues, which was consistent with the previous study of Won et al. [19]. Of 166 osteosarcoma specimens, 120 (72.3%) highly expressed SOX9. Then, we analyzed the associations of SOX9 expression with various clinicopathological parameters of osteosarcoma tissues. As shown in Table 1, high SOX9 expression was more frequently occurred in osteosarcoma tissues with advanced clinical stage (P = 0.02), positive distant metastasis (P = 0.008) and poor response to chemotherapy (P = 0.02). No significant difference was observed between the expression of SOX9 and patients’ age, gender, tumor size, anatomic location, serum levels of lactate dehydrogenase and alkaline phosphatase, and response to chemotherapy.

### Upregulation of SOX9 protein associates with poor prognosis in patients with osteosarcomas

**Figure 2:**
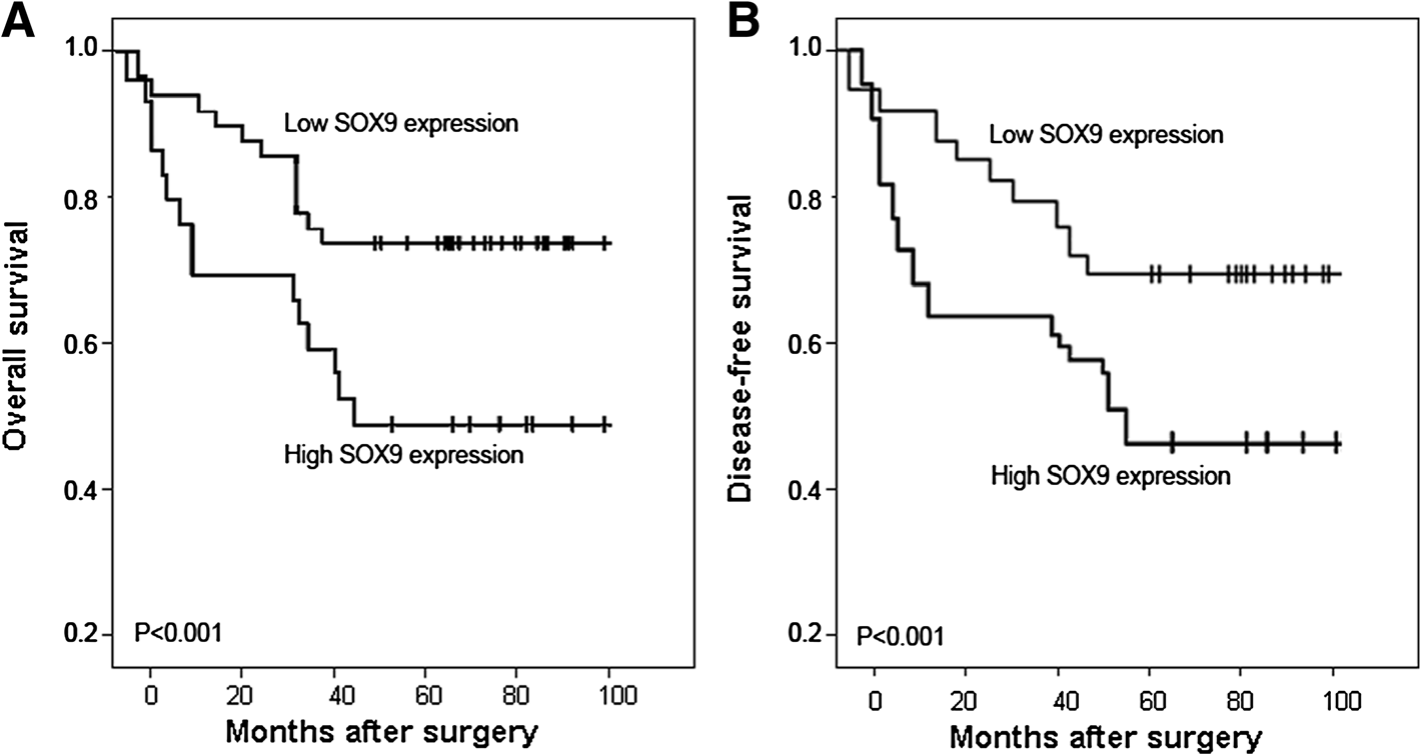
**Kaplan–Meier curves for patients with osteosarcoma.** Overall survival **(A)** and disease-free survival **(B)** curves for two groups defined by low and high expression of SOX9 in patients with osteosarcoma. The patients with high SOX9 expression had a significantly worse 5-year overall survival and disease-free survival rates than those with low SOX9 staining (both P < 0.001).

Using Kaplan–Meier method and log-rank test, the overall survival (OS, Figure 2A, P < 0.001) and disease-free survival (DFS, Figure 2B, P < 0.001) of osteosarcoma tissues with high SOX9 expression were both significantly shorter than those with low SOX9 expression (both P < 0.001). Besides, the survival benefits were also found in those with smaller tumor size (both P = 0.03), higher clinical stage (P = 0.01 and 0.006, respectively), without distant metastasis (P = 0.008 and 0.003, respectively), and better response to chemotherapy (both P = 0.02) for OS and DFS.

Multivariate Cox regression analysis enrolling above-mentioned significant parameters revealed that SOX9 expression (RR 6.9, 95% CI, 1.5-14.8 P < 0.001), clinical stage (RR 2.3, 95% CI, 1.1–7.0, P = 0.02), distant metastasis status (RR 3.7, 95% CI, 1.9–9.8, P = 0.01), and response to chemotherapy (RR 2.6, 95% CI, 1.5–8.2, P = 0.02) were independent prognostic markers for OS of patients with osteosarcoma (Table 2). Turning to DFS, SOX9 expression (RR 6.1, 95% CI, 1.3-13.9, P < 0.001), clinical stage (RR 1.8, 95% CI, 0.7–8.1, P = 0.03) and metastasis status (RR 2.9, 95% CI, 1.3–10.6, P = 0.02) were also independent prognostic markers for DFS of patients with osteosarcoma (Table 2).

**Table 2:** Multivariate survival analysis of OS and DFS in 166 patients with osteosarcoma

**Variables**	**OS**			**DFS**		
	**RR**	**95% CI**	**P**	**RR**	**95% CI**	**P**
**SOX9 expression**	6.9	1.5-14.8	<0.001	6.1	1.3-13.9	<0.001
**Clinical stage**	2.3	1.1-7.0	0.02	1.8	0.7-8.1	0.03
**Distant metastasis status**	3.7	1.9-9.8	0.01	2.9	1.3-10.6	0.02
**Response to chemotherapy**	2.6	1.5-8.2	0.02	0.9	0.3-1.7	0.6

## Discussion

Many molecular markers have proven prognostic value in osteosarcoma. It would be helpful if these markers were used as objective instruments for predicting the chance of survival or chemotherapy response, especially early in treatment, preferably even before surgery. The major finding of our study is that the upregulation of SOX9 expression is associated with the progression of osteosarcoma. To the best of our knowledge, the current study represents the first demonstration that SOX9 mRNA and protein are upregulated in osteosarcoma and that this degree of upregulation promotes advanced tumor progression.

The SOX gene family, which encodes transcription factors that bind to high-mobility group domains of DNA, has been found to share homology with the HMG box of the SRY [20]. Members of this family play critical roles in embryonic development, cell fate determination, differentiation, and proliferation [21]. SOX9, together with SOX8 and Sox10, belongs to the subgroup of the SOX E family [22]. It was originally known as a chondrogenic transcription factor involved in bone formation and testis development, whereas its mutations are the cause of the human disease campomelic dysplasia, a form of dwarfism characterized by extreme cartilage and bone malformation, which is frequently associated with XY sex reversal [23]. Since it is well recognized that genes and pathways critical for development may also play important roles in cancer development and progression, it is not surprising that SOX9 is involved in cancers. Zhou et al. [9] reported that the elevated expression of SOX9 may be related with the progression of gastric carcinoma; Chakravarty et al. [10] demonstrated that cytoplasmic SOX9 may serve as a valuable prognostic marker for invasive ductal carcinomas and metastatic breast cancer, and its significant correlation with breast tumor cell proliferation implied that SOX9 may directly contribute to the poor clinical outcomes associated with invasive breast cancer; Vidal et al. [11] found that SOX9 expression was a general feature of basal cell carcinoma and adnexal skin neoplasms; Lü et al. [12] suggested that SOX9 expression may be upregulated in colorectal cancer and strong SOX9 expression may be an independent adverse prognosticator in this cancer; Huang et al. [13] indicated that Sox9 may be required for prostate development and prostate cancer initiation; Zhou et al. [14] reported that SOX9 upregulation may be an independent prognostic indicator for the survival of patients with non-small cell lung cancer; Tanaka et al. [15] also found that SOX9 might contribute to carcinogenesis in pancreatic ductal adenocarcinoma and intraductal papillary mucinous neoplasm. In contrast, Passeron et al. [17] analyzed SOX9 expression in melanoma and discovered that SOX9 expression gradually decreased according to disease progress from normal skin to nevi, primary melanoma, and metastatic melanoma. Together, these observations suggest that SOX9 may be an attractive candidate marker that is involved in the tumor initiation and tumor progression, and it may play different roles depending on cancer types.

In the current study, there are four points of our findings. At first, SOX9 was up-regulated in human osteosarcoma tissues compared with noncancerous bone tissues at both mRNA and protein levels. However, Won et al. [19] in 2009 produced 48 formalin-fixed, paraffin-embedded tissue microarrays containing osteosarcoma tissue cores for immunohistochemical staining of SOX9. They observed negative immunostaining of SOX9 in 37 (77.1%) cores. The discrepancy between our data and this previous study may be caused by two reasons: (1) this previous study included only a small number of osteosarcoma tissues; (2) the heterogenicity of osteosarcoma tissues included in different studies. Secondly, the increased SOX9 expression in osteosarcoma tissues was significantly correlated with aggressive clinicopathological features; Thirdly, the results of Kaplan-Meier analyses shown that osteosarcoma tissues with high SOX9 expression tend to have shorter overall survival and disease-free survival. Finally, the multivariate analysis clearly demonstrated that SOX9 overexpression was a statistically significant risk factor affecting both overall survival and disease-free survival in osteosarcoma patients, suggesting that SOX9 expression could be a valuable marker of tumor progression and prognosis of osteosarcoma.

## Conclusions

In conclusion, our data show for the first time that SOX9 is upregulated in aggressive osteosarcoma tissues indicating that SOX9 may participate in the tumor progression of osteosarcoma. More importantly, SOX9 status is a useful prognostic factor for predicting the prognosis of osteosarcoma, suggesting that SOX9 may contribute to the optimization of clinical treatments for osteosarcoma patients.

## Competing interest

The authors declare that they have no competing interest.

## Authors' contributions

Haibo Zhu and Haikang Cai designed the study and drafted the manuscript; Haibo Zhu, Jie Tang, Mingjie Tang carried out the expertiments and performed the data analysis. All authors read and approved the final manuscript.

## References

[B1] Gill J Ahluwalia MK Geller D Gorlick R New targets and approaches in osteosarcoma Pharmacol Ther 2013 137 89 99 22983152 10.1016/j.pharmthera.2012.09.003

[B2] Bielack SS Kempf-Bielack B Delling G Exner GU Flege S Helmke K Kotz R Salzer-Kuntschik M Werner M Winkelmann W Zoubek A Jürgens H Winkler K Prognostic factors in high-grade osteosarcoma of the extremities or trunk: an analysis of 1,702 patients treated on neoadjuvant cooperative osteosarcoma study group protocols J Clin Oncol 2002 20 776 790 11821461 10.1200/JCO.2002.20.3.776

[B3] Jaffe N Jaffe N, Bielack SS, Bruland OS Adjuvant chemotherapy in osteosarcoma: An odyssey of rejection and vindication Pediatric and Adolescent Osteosarcoma, Cancer Treatment and Research 2009 New York: Springer 152 10.1007/978-1-4419-0284-9_11 20213393

[B4] Bakhshi S Radhakrishnan V Prognostic markers in osteosarcoma Expert Rev Anticancer Ther 2010 10 271 287 20132002 10.1586/era.09.186

[B5] Magnan H Chou AJ Chou JF Yeung HW Healey JH Meyers PA Noninvasive imaging with thallium-201 scintigraphy may not correlate with survival in patients with osteosarcoma Cancer 2010 116 4147 4151 20564163 10.1002/cncr.25375

[B6] Jakob S Lovell-Badge R Sex determination and the control of Sox9 expression in mammals FEBS J 2011 278 1002 1009 21281448 10.1111/j.1742-4658.2011.08029.x

[B7] Barrionuevo F Scherer G SOX E genes: SOX9 and SOX8 in mammalian testis development Int J Biochem Cell Biol 2010 42 433 436 19647095 10.1016/j.biocel.2009.07.015

[B8] Gordon CT Tan TY Benko S Fitzpatrick D Lyonnet S Farlie PG Long-range regulation at the SOX9 locus in development and disease J Med Genet 2009 46 649 656 19473998 10.1136/jmg.2009.068361

[B9] Zhou CJ Guo JQ Zhu KX Zhang QH Pan CR Xu WH Wang HJ Liu B Elevated expression of SOX9 is related with the progression of gastric carcinoma Diagn Cytopathol 2011 39 105 109 20301211 10.1002/dc.21348

[B10] Chakravarty G Moroz K Makridakis NM Lloyd SA Galvez SE Canavello PR Lacey MR Agrawal K Mondal D Prognostic significance of cytoplasmic SOX9 in invasive ductal carcinoma and metastatic breast cancer Exp Biol Med (Maywood) 2011 236 145 155 21321311 10.1258/ebm.2010.010086

[B11] Vidal VP Ortonne N Schedl A SOX9 expression is a general marker of basal cell carcinoma and adnexal-related neoplasms J Cutan Pathol 2008 35 373 379 18333897 10.1111/j.1600-0560.2007.00815.x

[B12] Lü B Fang Y Xu J Wang L Xu F Xu E Huang Q Lai M Analysis of SOX9 expression in colorectal cancer Am J Clin Pathol 2008 130 897 904 19019766 10.1309/AJCPW1W8GJBQGCNI

[B13] Huang Z Hurley PJ Simons BW Marchionni L Berman DM Ross AE Schaeffer EM Sox9 is required for prostate development and prostate cancer initiation Oncotarget 2012 3 651 663 22761195 10.18632/oncotarget.531 PMC3442290

[B14] Zhou CH Ye LP Ye SX Li Y Zhang XY Xu XY Gong LY Clinical significance of SOX9 in human non-small cell lung cancer progression and overall patient survival J Exp Clin Cancer Res 2012 31 18 22385677 10.1186/1756-9966-31-18 PMC3313873

[B15] Tanaka T Kuroki T Adachi T Ono S Hirabaru M Soyama A Kitasato A Takatsuki M Hayashi T Eguchi S Evaluation of SOX9 expression in pancreatic ductal adenocarcinoma and intraductal papillary mucinous neoplasm Pancreas 2013 42 488 493 23146920 10.1097/MPA.0b013e318269d281

[B16] Wang L He S Yuan J Mao X Cao Y Zong J Tu Y Zhang Y Oncogenic role of SOX9 expression in human malignant glioma Med Oncol 2012 29 3484 3490 22714060 10.1007/s12032-012-0267-z

[B17] Passeron T Valencia JC Namiki T Vieira WD Passeron H Miyamura Y Hearing VJ Upregulation of SOX9 inhibits the growth of human and mouse melanomas and restores their sensitivity to retinoic acid J Clin Invest 2009 119 954 963 19273910 10.1172/JCI34015 PMC2662541

[B18] Tang J Cai H Lin L Xie P Zhong W Tang M Increased expression of CD24 is associated with tumor progression and prognosis in patients suffering osteosarcoma Clin Transl Oncol 2013 15 541 547 23143956 10.1007/s12094-012-0961-5

[B19] Won KY Park HR Park YK Prognostic implication of immunohistochemical Runx2 expression in osteosarcoma Tumori 2009 95 311 316 19688969 10.1177/030089160909500307

[B20] Sarkar A Hochedlinger K The sox family of transcription factors: versatile regulators of stem and progenitor cell fate Cell Stem Cell 2013 12 15 30 23290134 10.1016/j.stem.2012.12.007 PMC3608206

[B21] Kawaguchi Y Sox9 and programming of liver and pancreatic progenitors J Clin Invest 2013 123 1881 1886 23635786 10.1172/JCI66022 PMC3635727

[B22] Knower KC Kelly S Harley VR Turning on the male–SRY, SOX9 and sex determination in mammals Cytogenet Genome Res 2003 101 185 198 14684982 10.1159/000074336

[B23] Castillo SD Sanchez-Cespedes M The SOX family of genes in cancer development: biological relevance and opportunities for therapy Expert Opin Ther Targets 2012 16 903 919 22834733 10.1517/14728222.2012.709239

